# Adaptive evolution of *Salmonella* Typhimurium LT2 exposed to carvacrol lacks a uniform pattern

**DOI:** 10.1007/s00253-023-12840-6

**Published:** 2024-01-04

**Authors:** Elisa Pagan, Natalia Merino, Daniel Berdejo, Raul Campillo, Elisa Gayan, Diego García-Gonzalo, Rafael Pagan

**Affiliations:** Departamento de Producción Animal y Ciencia de los Alimentos, Facultad de Veterinaria, Instituto Agroalimentario de Aragón-IA2, Universidad de Zaragoza-CITA, Zaragoza, Spain

**Keywords:** Essential oils, Antibiotics, Adaptive laboratory evolution, *Salmonella* Typhimurium, Whole genome sequencing, Cross resistance

## Abstract

**Abstract:**

Emergence of genetic variants with increased resistance/tolerance to natural antimicrobials, such as essential oils, has been previously evidenced; however, it is unknown whether mutagenesis follows a general or a specific pattern. For this purpose, we carried out four adaptive laboratory evolutions (ALE) in parallel of *Salmonella enterica* Typhimurium with carvacrol. After 10 evolution steps, we selected and characterized one colony from each lineage (SeCarA, SeCarB, SeCarC, and SeCarD). Phenotypic characterization of the four evolved strains revealed enhanced survival to lethal treatments; two of them (SeCarA and SeCarB) showed an increase of minimum inhibitory concentration of carvacrol and a better growth fitness in the presence of carvacrol compared to wild-type strain. Whole genome sequencing revealed 10 mutations, of which four (*rrsH*, *sseG*, *wbaV*, and *flhA*) were present in more than one strain, whereas six (*nirC*, *fliH*, *lon*, *rob*, upstream *yfhP*, and upstream *argR*) were unique to individual strains. Single-mutation genetic constructs in SeWT confirmed *lon* and *rob* as responsible for the increased resistance to carvacrol as well as to antibiotics (ampicillin, ciprofloxacin, chloramphenicol, nalidixic acid, rifampicin, tetracycline, and trimethoprim). *wbaV* played an important role in increased tolerance against carvacrol and chloramphenicol, and *flhA* in cross-tolerance to heat treatments. As a conclusion, no common phenotypical or genotypical pattern was observed in the isolated resistant variants of *Salmonella* Typhimurium emerged under carvacrol stress. Furthermore, the demonstration of cross-resistance against heat and antibiotics exhibited by resistant variants raises concerns regarding food safety.

**Key points:**

*• Stable resistant variants of Salmonella Typhimurium emerged under carvacrol stress*

*• No common pattern of mutagenesis after cyclic exposures to carvacrol was observed*

*• Resistant variants to carvacrol showed cross-resistance to heat and to antibiotics*

**Graphical abstract:**

**Figure:**
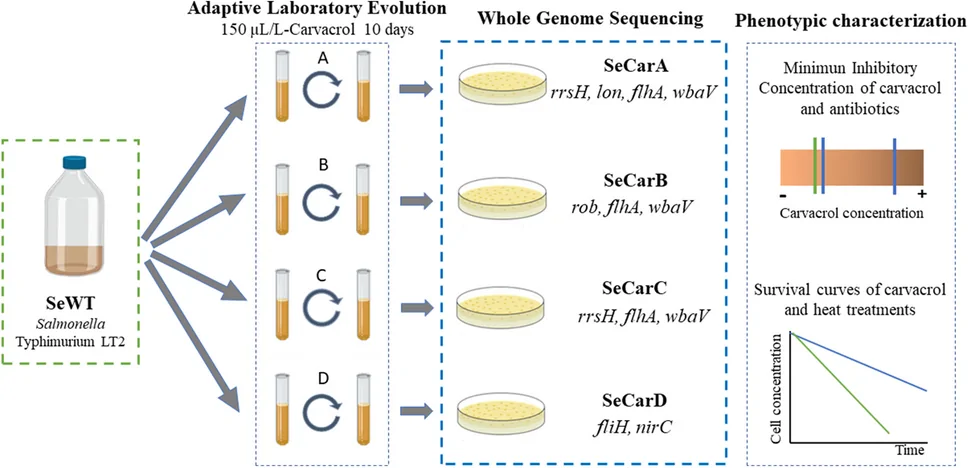

**Supplementary Information:**

The online version contains supplementary material available at 10.1007/s00253-023-12840-6.

## Introduction

Antimicrobial resistance (AMR) has recently become one of the most acute international public health issues, as it threatens the effective prevention and treatment of infections resulting from a wide range of pathogens (Zhang et al. 2022). One approach to analyze the emergence and the mode of action of de novo mutations conferring antibiotic resistance is the study of adaptive laboratory evolution (ALE) (Lopatkin et al. 2022). The ALE process involves the continuous culturing of a microorganism over multiple generations under specific conditions. During an ALE experiment, a series of mutations arise, and those that are prone to help withstand selection pressure are fixed over time in the population. Two of the main bacterial adaptation strategies toward antibiotics are resistance and tolerance (Brauner et al. 2016). “Resistance” is used to describe the inherited ability of microorganisms to grow at high concentrations of an antibiotic, irrespective of the duration of treatment, and is quantified by the minimum inhibitory concentration (MIC) or by the study of a microorganism’s growth curve kinetics. “Tolerance,” on the other hand, describes the ability of a population to survive transient exposure to a high dose of an antibiotic. A tolerant population does not necessarily have a higher MIC, but it exhibits a slower killing rate compared to a susceptible population (Fridman et al. 2014; Levin-Reisman et al. 2017).

Once a microbial population has been subjected to an ALE procedure, a more comprehensive view of resistance mechanisms can be obtained by attempting to understand which genetic changes enabled an increase in resistance or tolerance: this can be achieved by directly linking genes to the phenotype (Dettman et al. 2012; Jahn et al. 2017; Sulaiman and Lam 2021). Apart from the study of microbial resistance to antibiotics, evolution experiments have been carried out with the purpose of elucidating other mechanisms of adaptation to stresses that can be induced during food preservation, such as high temperature (Gayan et al. 2016; Sandberg et al. 2014), high hydrostatic pressure (Vanlint et al. 2012), and acid medium (Harden Mark et al. 2015).

In addition to the problem caused by AMR, the reduced popularity of synthetic compounds among consumers has increased the general of interest in natural antimicrobial agents as an alternative (Dini et al. 2022). Essential oils (EOs) and their individual constituents (ICs) have demonstrated excellent antimicrobial as well as antioxidant properties (Quinto et al. 2019), and have been proposed as an alternative to antibiotics in animal production (Zhang et al. 2022), as well as to conventional preservatives in the food industry (Leonelli et al. 2022; Mantzourani et al. 2022).

To explain the antimicrobial activity of EOs, several mechanisms of action have been proposed, including changes in the cell membrane fatty acid profile, damages to the cytoplasmic membrane, reduction of the cell’s proton-motive force, or the formation of reactive oxygen species (ROS) (Chueca et al. 2014). However, further studies are required to understand the mechanisms of these compounds at a molecular level (Rao et al. 2019).

In two of our previous studies, we isolated the following strains by cyclic exposure to prolonged sublethal treatments as well as to short lethal treatments: 1) resistant and tolerant strains of *S.* Typhimurium against to carvacrol (Berdejo et al. 2020a), and 2) a strain resistant and tolerant to *Thymbra capitata* EO (TCO), of which carvacrol is the main component. Berdejo et al. (2021a) demonstrated that the amino acid replacement of arginine by leucine at position 20, specifically in the DNA-binding domain of *soxR*, a redox-sensitive transcriptional activator, was the main cause of the increased resistance and tolerance observed against TCO. Since those studies were carried out on one sole evolved population, and since the evolutionary path leading to a final population genotype can be highly variable and unpredictable, a great deal of information regarding the mechanism of action of carvacrol may still be lacking. In this regard, mutations that emerge in populations subjected to parallel carvacrol ALE under the exact same conditions can turn out to be different, as Lopatkin et al. (2022) described for a series of antibiotics.

For this reason, the aim of this study was a) to identify and compare all the genetic modifications obtained from different lineages of carvacrol ALE with the purpose of understanding whether the mutagenesis follows a general or a specific pattern, b) to determine which mutations are responsible for the increase in bacterial resistance by single-mutation genetic constructs, and c) to study the occurrence of cross-resistance/tolerance in the evolved strains to antibiotics and food preservation technologies.

## Materials and methods

### Microorganisms and growth conditions

*Salmonella enterica* subsp. *enterica* serovar Typhimurium LT2 (SeWT) was provided by the Spanish Type Culture Collection (CECT 722). SeWT and the evolved strains obtained in this study were maintained in cryovials at – 80 °C with glycerol (20% v/v), from which plates of tryptone soya agar (Oxoid, Basingstoke, England) with 0.6% yeast extract (Oxoid; TSAYE) were prepared on a weekly basis. To prepare overnight precultures, tubes containing 5 mL of fresh tryptone soya broth (Oxoid) with 0.6% yeast extract (TSBYE) were inoculated with one colony and incubated aerobically in an orbital shaker (130 rpm; Heidolph Vibramax 100, Schwaback, Germany) for 12 h at 37 °C (Incubig, Selecta, Barcelona, Spain). Subsequently, working bacterial cultures were prepared in flasks containing 10 mL of TSBYE, which were inoculated with an initial concentration of 10^6^ colony forming units per mL (CFU/mL) from the preculture and incubated for 24 h at 37 °C under 130 rpm until the stationary growth phase (5 × 10^9^ CFU/mL approximately) was reached. The cultures’ bacterial concentration was verified by diluting them in phosphate-buffered saline (Sigma-Aldrich; PBS) and spreading on TSAYE plates. The same protocol was applied to obtain the working bacterial cultures of the evolved strains that resulted from the ALE experiments with carvacrol.

### Minimum inhibitory concentration (MIC) of carvacrol

Following the method described by Friedman et al. (2002), a vigorous shaking by vortex (Genius 3, Ika, Königswinter, Germany) was used to prepare carvacrol dispersions in 5 mL of TSBYE at different concentrations: from 50 up to 500 µL/L, avoiding the use of solvents in view of their possible detrimental effect on antibacterial activity. Positive controls with only bacteria and negative controls with only carvacrol were prepared in each experiment. The strains were inoculated at 5 × 10^5^ CFU/mL in TSBYE and incubated at 37 °C for 24 h. MIC was determined as the lowest concentration of the antimicrobial compound capable of inhibiting bacterial growth after incubation. Bacterial growth was measured by optical density with a microplate reader (Genios, Tecan, Mannedorf, Switzerland) at 595 nm (OD_595_ nm).

### Carvacrol ALE in parallel

The ALEs in this study were based on the isolation of strains by prolonged exposure to a subinhibitory concentration of carvacrol during bacterial growth. This procedure was carried out four times in parallel: one per lineage (A–D). SeWT was grown on TSAYE plates for 24 h at 37 °C. A single colony was inoculated in 5-mL TSBYE and incubated under agitation for 12 h at 37 °C. This preculture was diluted 1:1000 into 50-mL TSBYE and incubated for 3.5 h to obtain an exponential growth phase culture. From that culture, SeWT were inoculated at an initial bacterial concentration of 10^6^ CFU/mL in 5 mL TSBYE with 100 µL/L of carvacrol (1/2 × MIC). This bacterial suspension was incubated 24 h/37 °C/130 rpm and, once stationary phase was reached, the same dilution steps were repeated 10 times: the culture was inoculated (10^6^ CFU/mL) in 5-mL TSBYE with 100 µL/L of carvacrol and incubated 24 h/37 °C/130 rpm. After the 10th step, an aliquot was diluted in PBS and spread on TSAYE plates. After the incubation period, up to ten colonies from each lineage were randomly selected. The first approach to evaluate their resistance was to determine the MIC of carvacrol in comparison to that in SeWT, after which the strain with the highest MIC from each lineage was genotypically characterized. Selected evolved strains from each lineage (A–D) were named “SeCarA,” “SeCarB,” “SeCarC,” and “SeCarD.”

### Growth curves in presence of carvacrol

In order to further evaluate the resistance capacity of the evolved strains (SeCarA, SeCarB, SeCarC, SeCarD), their growth kinetics and that of SeWT were evaluated in TSBYE in absence of as well as in the presence of 100 µL/L of carvacrol. First, carvacrol was added to tubes with 5 mL of TSBYE. Due to the hydrophobicity of carvacrol, it was necessary to apply vigorous agitation in vortex to obtain a uniform suspension. Once carvacrol was added, test tubes were inoculated with the bacterial culture at an initial concentration of 5 × 10^5^ CFU/mL and incubated at 37 °C and 130 rpm for 24 h. Each hour, OD_595_ of the test tubes was measured by a microplate reader. A positive control (without antimicrobial added) and a negative control (without microbial culture added) were included in all the assays. The values of OD_595_ at time 0 corresponding to the absorbance caused by the growth medium were subtracted. Bacterial growth curves based on OD_595_ of SeWT, SeCarA, SeCarB, SeCarC, and SeCarD were graphically displayed and modeled by modified Gompertz equation:

1 $${\mathrm{y}}=A {\mathrm{exp}}\left\{- exp\left[ ({\mu }_{m} e /A )\left(\lambda - t\right)+1\right]\right\}$$

where *y*: OD_595_; *t*: time (h); *A*: maximum value reached (OD_595_ max); *µ*_*m*_: maximum specific growth rate (h^−1^); *λ*: lag time (h).

A least-squares adjustment was carried out to build the model and to obtain *A*, *µ*_*m*_, and *λ* values using the GraphPrism® program (GraphPad Software, Inc., San Diego, USA). The adjustment’s goodness of fit was evaluated using standard error, *R*^2^, and *R*^2^ adjusted values, as well as the root mean square error (RMSE).

### Survival curves in presence of carvacrol

The tolerance of SeWT and of the evolved strains to lethal treatments was likewise evaluated. The treatment medium we used in these assays was “McIlvaine buffer” (citrate–phosphate buffer), prepared from citric acid monohydrate (Panreac), and disodium hydrogen phosphate (Panreac), adjusted to pH 7.0. Carvacrol treatment was performed in 10-mL McIlvaine buffer previously tempered to 25 °C, to which carvacrol was added at a concentration of 150 µL/L and then vigorously agitated to obtain a homogeneous dispersion. Stationary-phase cultures were centrifuged for 3 min at 10,000 RCF in a microcentrifuge (Mini Spin, Eppendorf, Hamburg, Germany) and resuspended in the treatment medium. Test tubes were inoculated at 10^7^ CFU/mL and aliquots were obtained every 5 min during 30 min in total. The treated samples for each assay were diluted in PBS and subsequently spread on TSAYE plates. After plate incubation (24 h/37 °C), the count of survival cells was carried out in an automatic plate counter by image analysis (Analytical Measuring Systems, Protos, Cambridge, UK).

### Whole genome sequencing (WGS) and identification of genetic variations

Genomic DNA (gDNA) of SeWT and of evolved strains was extracted and purified using a gDNA kit (DNeasy kit, Qiagen, Hilden, Germany). Illumina technology was used to carry out whole genome sequencing (WGS) on NextSeq equipment at mid-output flow, with a total of 2 × 150 cycles (Illumina; Novogene Co. Ltd., UK). Subsequently, Novogene performed the bioinformatic analysis. The quality-control-filtered paired-end reads were mapped on the reference genome sequence (National Center for Biotechnology Information; NCBI accession: NC_003197.2): *Salmonella enterica* subsp. *enterica* serovar Typhimurium str. LT2, complete genome, using a Burrows-Wheeler Alignment (BWA) tool [31] and Samtools software (sources: http://bio-bwa.sourceforge.net and http://www.htslib.org). Raw-coverage 350-fold depth was achieved for all five strains. Samtools was then applied to remove potential PCR duplicates according to reading positions on the reference genome. To correct mapping errors and insert the quality values, the resulting BAM files were further processed using LoFreq-Star (source: http://csb5.github.io/lofreq). Finally, single-nucleotide variants (SNVs) and short insertions and deletions (InDels) were detected using LoFreq-Star, and the snpEff toolbox (source: http;//snpeff.sourceforge.net) was employed to identify involved genes and to predict functional effect variations. Coverage was further analyzed by the Integrative Genomics Viewer (IGV; Broad Institute, source: https://software.broadinstitute.org/software/igv) in order to find structural variations (SVs). Although mapping was carried out against the reference genome, SNVs, InDels, and SVs were identified between SeWT and evolved strains to ascertain the kind of mutations that had occurred during the ALE. Finally, specific primers (Table S1) were designed with the NCBI primer designing tool in order to carry out PCR amplifications, as well as Sanger sequencings to verify the mutations detected in the WGS. The resulting genome sequences were deposited in the Sequence Read Archive (SRA) of NCBI (BioProject ID: PRJNA951735). The accession numbers of the samples are SAMN34055370 (SeWT), SAMN34055371 (SeCarA), SAMN34055372 (SeCarB), SAMN34055373 (SeCarC), and SAMN34055374 (SeCarD). Additionally, Table S2 summarizes the genomic background of* S*. Typhimurium LT2.

### Strain construction

In order to perform the mutated gene replacement of *lon*, *rob*, *nirC*, *wbaV*, and *yfhP* in SeWT, red recombinase technology was applied. The XTL298 strain, which contains *tetA*-*sacB* in the arabinose operon for strain construction, was kindly provided by Donald L. Court (National Cancer Institute at Frederick, MD, USA). The *tetA*-*sacB* cassette (Li et al. 2013) was used as the PCR template to generate DNA fragments for primary chromosomal integration near to mutated gene using specific primers containing 50 base pair (bp) homologous sequences (Table S2). Plasmid pKD46, encoding the λ red recombinase genes behind the *araBAD* promoter, was used to enable the chromosomal integrations via double-crossover recombination, as previously described by Datsenko and Wanner (2000). To replace wild-type allele by gene variant in SeWT using intrachromosomal recombination, we first marked wild-type allele with *tetA*-*sacB* cassette. Then the *tetA*–*sacB* cassette of this strain was replaced intrachromosomally by gene variant with the aid of λ-red recombinase.

Gene deletions of *flhA* and *fliH* were performed according to the method propounded by Datsenko and Wanner (2000). An amplicon prepared on pKD13 (containing the kanamycin resistance cassette [nptI]) with the primers listed in Table S2 was recombined in-frame after the start codon of the target gene of a pKD46-equipped strain.

LB broth and agar were routinely used for strain and plasmid construction; moreover, when necessary, a final concentration of either 50 μg/mL of kanamycin (Panreac-AppliChem, Darmstadt, Germany), 20 μg/mL of tetracycline (Sigma-Aldrich, St. Louis, MO, USA), 100 μg/mL of ampicillin (Thermo Fisher Scientific), or 1.5% (w/v) sucrose was added to select for the presence of plasmids or recombined amplicons. PCR and Sanger sequencing were used to verify the replacement of the mutant allele in the SeWT strain. Once the new strains with the mutated genes had been obtained, MIC of carvacrol, survival curves, and MIC of antibiotics were obtained against them following the procedures described above.

### Characterization of cross-resistance/tolerance against heat and antibiotics

For thermal treatment, the procedure described above was followed. In this case, PCR tubes were each aseptically filled with 60 μL of resuspended cells in McIlvaine buffer pH 7.0 (10^9^ CFU/mL) and subjected to heat (54 °C for 25 min) using a PCR apparatus (T100 Thermal Cycler, Bio-rad, Spain).

MIC determinations of antibiotics were evaluated in Mueller Hinton broth cation adjusted (Sigma-Aldrich; MHB) according to CLSI. The tested antibiotics were ampicillin, cephalexin, ciprofloxacin, chloramphenicol, kanamycin, nalidixic acid, rifampicin, tetracycline, and trimethoprim. Standard antibiotics were serially diluted twofold across 96-well plates with final volumes of 100 µL per well.

### Statistical analysis

All phenotypic characterization results were obtained from at least three independent experiments carried out on different working days with different bacterial cultures. Growth curve parameters and survival curve graphics are displayed as the mean ± standard deviation, using the GraphPrism® program. Data were analyzed and submitted to comparison of averages using analysis of variance (ANOVA), followed by post-hoc Tukey test and *t*-tests with GraphPrism®, and differences were considered significant if *p* ≤ 0.05.

## Results

### Isolation of resistant strains obtained by four independent ALE assays under selective pressure of carvacrol

After 10 days of ALE with carvacrol, up to 10 colonies of *S.* Typhimurium from each lineage (A–D) were randomly selected to carry out phenotypic characterization. MIC determinations against SeWT and evolved strains were performed to compare their resistance to carvacrol. Not all the colonies in a lineage displayed the same degree of resistance to carvacrol, neither was this the case in colonies stemming from different lineages. Table 1 shows the highest MIC observed against the colonies evaluated within each lineage. One colony from lineage A, named SeCarA, exhibited a 50% increase in carvacrol resistance compared to SeWT: it was inhibited at 200 μL/L of carvacrol. Among all the evolved strains, the highest increase in resistance was detected in one of the colonies of lineage B (SeCarB), which required 350 μL/L of carvacrol in order to be inhibited. Conversely, in the C and D lineages, none of the tested colonies showed an increase in resistance to carvacrol; we thus randomly selected one colony from each of these two lineages (SeCarC and SeCarD) in order to pursue the study.

**Table 1:** Minimum inhibitory concentration (MIC; µL/L) of carvacrol for *Salmonella enterica* Typhimurium LT2 (SeWT) and the selected strains evolved: SeCarA, SeCarB, SeCarC, and SeCarD

Strains	SeWT	SeCarA	SeCarB	SeCarC	SeCarD
MIC (μL/L)	200	300	350	200	200

### Further evaluation of resistance by modeling growth kinetics under carvacrol stress

Growth kinetics studies were carried out in the absence and presence of 100 μL/L carvacrol to characterize the fitness of selected evolved strains (SeCarA, SeCarB, SeCarC, and SeCarD) in comparison with SeWT. Growth curves were modeled according to Gompertz equation (Eq. 1) by least-squares adjustment with excellent goodness of fit (Table S3).

As observed in Table 2, in the absence of carvacrol, all strains showed a similar lag phase (*λ*) (*p* > 0.05); however, when carvacrol was present in the growth medium, all the evolved strains displayed a shorter lag phase than SeWT. For instance, SeCarB showed a reduction of 2 h in lag phase in comparison to SeWT. Regarding growth rate (*μ*_*m*_), significant differences (*p* ≤ 0.05) were only observed between the growth rate of SeWT and those of SeCarA and SeCarB in the presence of carvacrol: the latter two strains grew faster than SeWT. Significant differences (*p* ≤ 0.05) were also observed in the maximum absorbance values (*A*) of the evolved strains with respect to SeWT, both in the presence and in the absence of carvacrol.

**Table 2:** *λ* (lag phase time), *µ*_*m*_ (maximum specific growth rate), and *A* (maximum OD_595_) parameters of the modified Gompertz model obtained from growth curves of *Salmonella* Typhimurium LT2 (SeWT) and strains evolved (SeCarA, SeCarB, SeCarC and SeCarD) in the presence (100 μL/L) and absence of carvacrol. Each value represents the mean ± standard deviation from three independent experiments

		Strains				
Carvacrol (μL/L)	Parameter	SeWT	SeCarA	SeCarB	SeCarC	SeCarD
0	*λ* (h)	3.038 ± 0.113	2.947 ± 0.227	2.665 ± 0.214	2.946 ± 0.096	2.959 ± 0.324
	*µ*_*m*_ (OD_595_/h)	0.170 ± 0.015	0.213 ± 0.041	0.177 ± 0.011	0.196 ± 0.012	0.277 ± 0.064
	*A* (OD_595_)	0.688 ± 0.027	0.789 ± 0.024*	0.805 ± 0.023*	0.820 ± 0.010*	0.812 ± 0.011*
100	*λ* (h)	5.543 ± 0.138	4.379 ± 0.352*	3.593 ± 0.078*	3.865 ± 0.285*	3.642 ± 0.297*
	*µ*_*m*_ (OD_595_/h)	0.178 ± 0.012	0.300 ± 0.070*	0.356 ± 0.035*	0.197 ± 0.019	0.203 ± 0.026
	*A* (OD_595_)	0.572 ± 0.005	0.566 ± 0.039	0.628 ± 0.008*	0.631 ± 0.010*	0.674 ± 0.017*

^*^Significantly different from SeWT (*p* ≤ 0.05)

### Evaluation of tolerance by survival curves against carvacrol

**Fig. 1:**
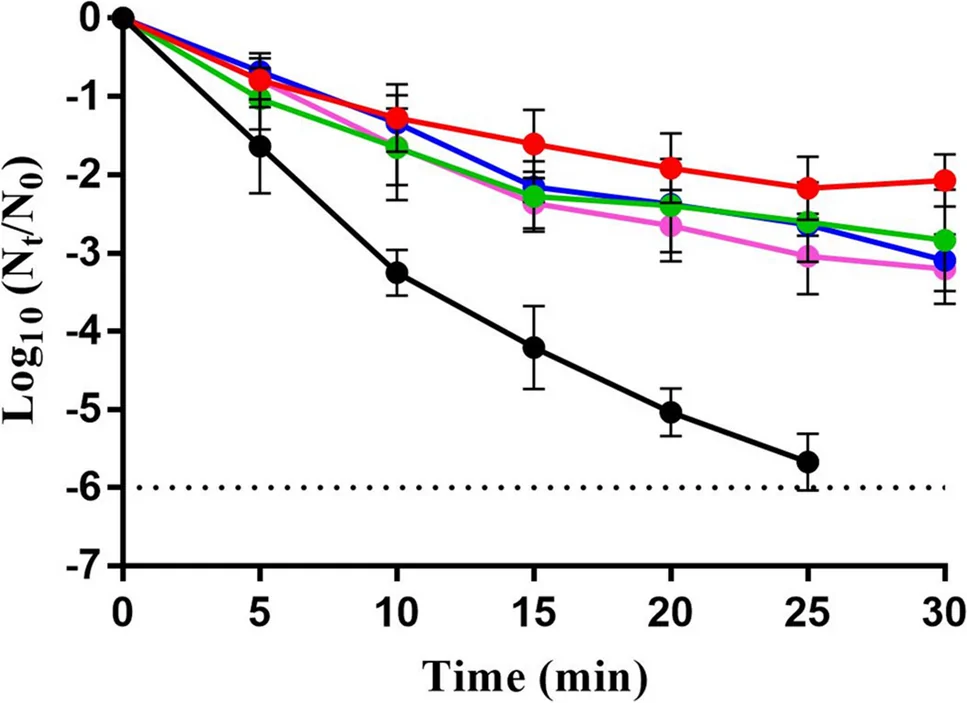
Survival curves of *Salmonella enterica* Typhimurium LT2 (●; SeWT) and its evolved strains, SeCarA (  ), SeCarB (  ), SeCarC (  ), and SeCarD (  ), exposed to 150-µL/L carvacrol treatment at pH 7.0. Data are means ± standard deviations (error bars) obtained from at least three independent experiments. The dashed line represents the detection limit (− 6 log_10_ *N*_t_/*N*_0_)

We further evaluated the tolerance of the evolved strains against lethal carvacrol treatments (150 µL/L) by comparing their survival curves with those of SeWT in McIlvaine buffer at pH 7.0 (Fig. 1). As can be seen in Fig. 1, the four evolved strains were more tolerant than SeWT: for instance, after 25 min of treatment, SeWT exhibited a reduction of more than 5.5 log_10_ cycles of inactivation, whereas the evolved strains only exhibited a reduction of approximately 2.5 log_10_ cycles. Notably, the inactivation kinetics were similar among the evolved strains (*p* > 0.05), indicating that the difference observed between the evolved strains and SeWT is due to tolerance rather than resistance. These findings highlight the distinction between resistance and tolerance, and they suggest that the evolved strains have acquired mechanisms that allow them to withstand transient exposure to high doses of carvacrol.

### Detection of genetic variations in evolved strains

The following step consisted in determining the genetic changes that contributed to the observed phenotypes. Genetic differences between the reference genome (NCBI accession: NC_003197.2) and our SeWT lab strain were firstly identified (Table S4). Genetic comparison between SeWT and evolved strains led to the identification of mutations fixed throughout the carvacrol ALE (Table 3). Of the 10 identified mutations, four were common SNVs found in multiple strains. For example, both SeCarA and SeCarC had the same SNV in *rrsH*, whereas SeCarA, SeCarB, and SeCarC all had SNVs in *sseG, wbaV*, and *flhA*. The remaining six SNVs were unique to individual strains. Table 3 provides information on the mutated genes thereby impacted, along with the expected changes resulting from them. Among the common SNVs, the following changes were found: a cytosine by an adenosine in *rrsH* or 16S rRNA ribosomal is the RNA component of the 30S small subunit of the ribosome; a silent mutation in *sseG* which codifies a secretor system effector; a replacement of tyrosine by serine in position 194 of the abequosyltransferase WbaV; and an amino acid substitution of proline by glycine at position 306 of the flagellar biosynthesis protein FlhA.

**Table 3:** Genetic variations of strains evolved by cyclic exposure to prolonged sublethal treatments of carvacrol (SeCarA, SeCarB, SeCarC, and SeCarD) in comparison with SeWT, verified by Sanger sequencing

Genome position	Strain	Genes	Locus tag	Mutation	Change	Information
290,742	SeCarA, SeCarC	*rrsH*	STM0249	SNV:C1553A	No coding	RNA 16S ribosomal
506,264	SeCarA	*lon*	STM0450	SNV: T722C	Leu241Pro	ATP-dependent protease
1,489,083	SeCarA, SeCarB, SeCarC	*sseG*	STM1405	SNV: T342C	Silent mutation (113Cys)	Secretor system effector
2,009,371	SeCarA, SeCarB, SeCarC	*flhA*	STM1913	SNV: C917A	Pro306Gln	Flagellar biosynthesis protein
2,059,229	SeCarD	*fliH*	STM1971	SNV: A30C	Thr11Pro	Flagellar assembly protein
2,168,037	SeCarA, SeCarB, SeCarC	*wbaV*	STM2087	SNV: T581C	Tyr194Ser	Abequosyltransferase
2,683,461	SeCarB	Upstream* yfhP*	upstream STM2544	SNV:C20T*	No coding	Hypothetical protein
3,626,869	SeCarD	*nirC*	STM3476	SNV: T215C	Val72Ala	Nitrite transporter
4,705,671	SeCarC	Upstream argR	upstream STM4463	SNV:C27A*	No coding	Arginine repressor
4,843,087	SeCarB	*rob*	STM4586	SNV: G467T	Arg156Leu	Transcriptional regulator

^*^From the coding region

*SNV*, single nucleotide variation

Regarding the unique SNVs, in SeCarA a substitution of leucine by proline at 241 position of the ATP-dependent serine Lon protease was detected; in SeCarB, arginine of position 156 is replaced by a leucine in *rob*, a transcriptional regulator; furthermore, in SeCarD, a replacement of threonine by proline in position 11 of FliH, related to flagellar assembly, and a missense mutation in *nirC*, resulting in a change from valine to alanine at position 72 of nitrite transporter protein NirC were detected.

Finally, two SNVs in intergenic regions were found: in SeCarC between STM4463 and STM4464 (encoding putative arginine repressor *argR*), and in SeCarB between *yfhP* and STM2545 (encoding hypothetical protein and putative rRNA methylase, respectively).

### Confirmation of genetic variations responsible for increased resistance and tolerance to carvacrol

**Fig. 2:**
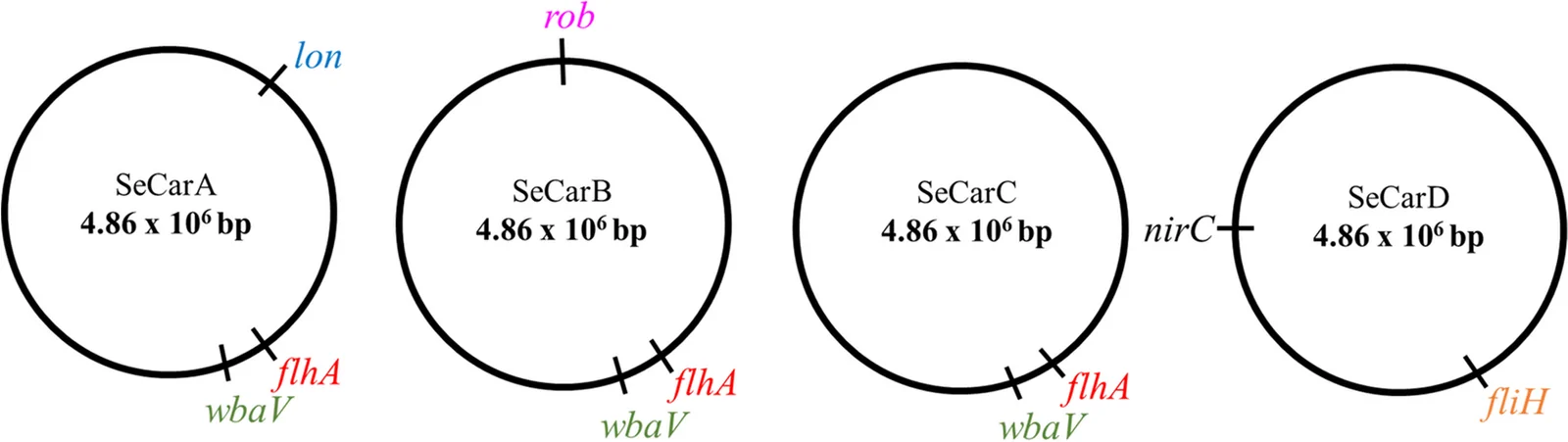
Genomic maps of the *Salmonella enterica* Typhimurium LT2 strains (SeCarA, SeCarB, SeCarC, and SeCarD) evolved by cyclic exposure to prolonged sublethal treatments of carvacrol

Once we had identified the genetic modifications in SeCarA, SeCarB, SeCarC, and SeCarD, based on the function of the mutated genes, we decided to focus our study on the *lon*, *rob*, *nirC*, *wbaV*, *yfhP*, *flhA*, and *fliH* mutations (Fig. 2). Thus, to determine the contribution of those single mutations to the phenotype of the evolved strains, we constructed Se_*lon*_, Se_*rob*_, Se_*nirC*_, Se_*wbaV*_, and Se_*yfhP*_ by replacing the desired gene in SeWT by the mutant alleles: *lon*^SeCarA^, *rob*^SeCarB^, *nirC*^SeCarD^, *wbaV*^SeCarA^, and *yfhP*^SeCarB^. Previous motility studies showed that the mutations found in *flhA* and *fliH* induced their loss of function; thus, for these genes, we chose to carry out gene deletions (∆*flhA* and ∆*fliH*) according to the method propounded by Datsenko and Wanner (2000).

Once the strains had been constructed, we applied MIC determination in order to compare their carvacrol resistance to that of the evolved strains. As shown in Table 4, the mutations found in *wbaV*, *yfhP*, and *flhA* were not responsible for the increased carvacrol resistance of SeCarA and SeCarB, since there were no differences between the MIC against their single-mutation genetic constructs and the MIC against the SeWT (200 µL/L). However, the mutations found in *lon* and *rob* could be attributed to increased resistance, since MIC of carvacrol for Se_*lon*_ was 300 μL/L, while for Se_*rob*_ it was 350 μL/L, which are the values obtained for SeCarA and SeCarB, respectively. As could be expected from the MIC values against the evolved strains SeCarC and SeCarD, their single-mutation genetic constructs (Se_*nirC*_, Se_*wbaV*_, Se_*yfhP*_, Se_*∆flhA*_, and Se_*∆fliH*_) did not cause any modification of the MIC values of carvacrol.

**Table 4:** Minimum inhibitory concentration (MIC; µL/L) of carvacrol for *Salmonella* Typhimurium LT2 (SeWT), the selected strains evolved (SeCarA, SeCarB, SeCarC, and SeCarD), and the constructed strains with isolated genes Se_*lon*_, Se_*rob*_, Se_*nirC*_, Se_*wbaV*_, Se_*yfhP*_, Se_∆*flhA*_, Se_∆*fliH*_

	MIC (μL/L)	Se_*lon*_	Se_*rob*_	Se_*nirC*_	Se_*wbaV*_	Se_*yfhP*_	Se_∆*flhA*_	Se_∆*fliH*_
SeWT	200							
SeCarA	300	300			200		200	
SeCarB	350		350		200	200	200	
SeCarC	200				200		200	
SeCarD	200			200				200

**Fig. 3:**
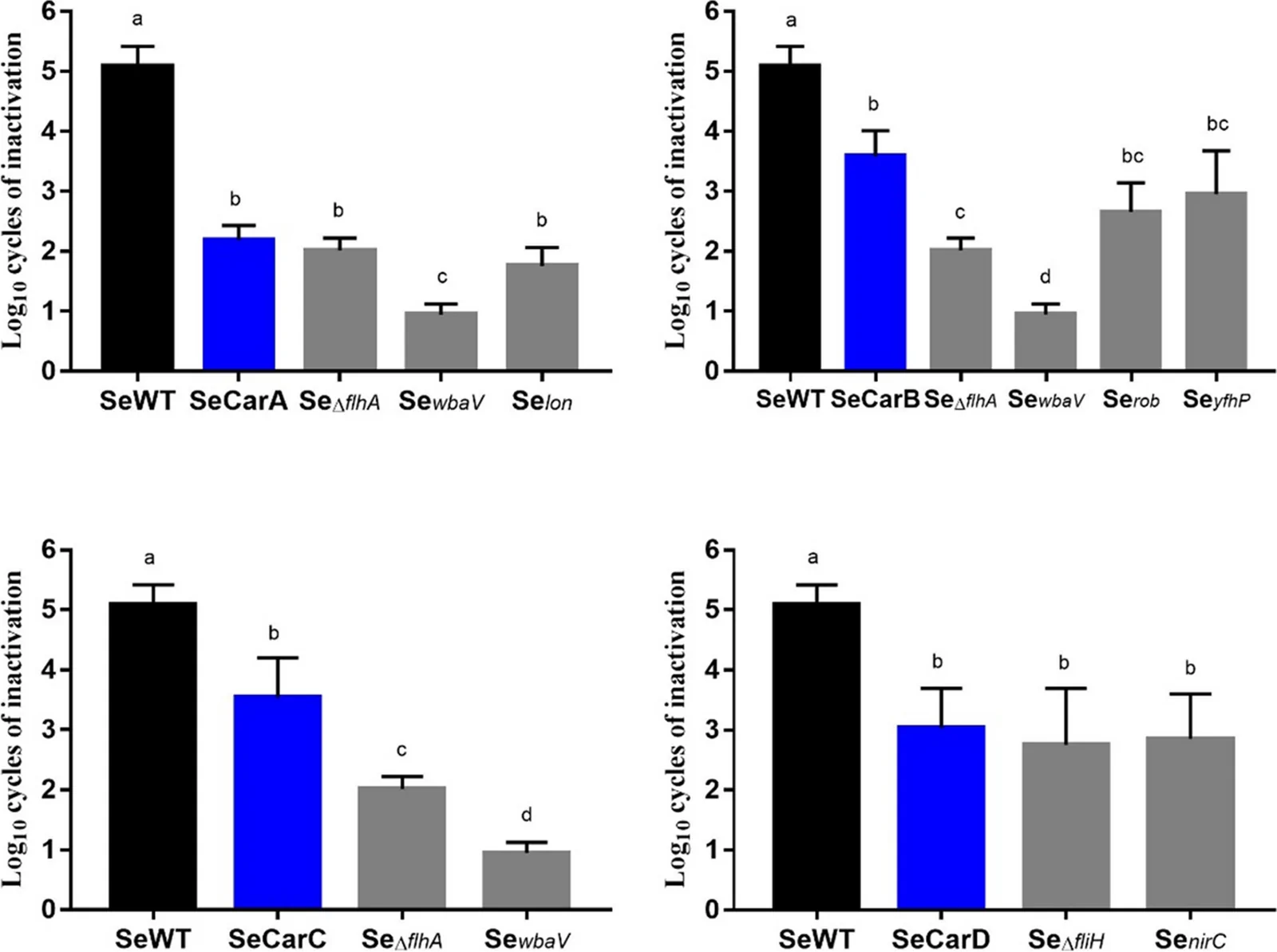
Log_10_ cycles of inactivation of *Salmonella enterica* Typhimurium LT2 (■; SeWT), the evolved strains (  ): SeCarA (A), SeCar (B), SeCarC (C), SeCarD (D), and the strains constructed with isolated genes (  ) after 20 min of 150 µL/L carvacrol treatment at room temperature. Data are means ± standard deviations (error bars) obtained from at least three independent experiments. Different superscript letters represent statistically significant differences (*p* ≤ 0.05)

The contribution of each identified SNV to bacterial tolerance against carvacrol was evaluated. Thus, SeWT, the evolved strains, and the constructed strains were all treated with 150 µL/L of carvacrol at pH 7.0 during 20 min. Results in Fig. 3 reveal significant differences among the inactivations obtained from the constructed strains and from SeWT; thus, all mutations found in the evolved strains were involved in direct tolerance against carvacrol. Specifically, Se_*nirC*_, Se_*lon*_, Se_*yfhP*_, and Se_*∆fliH*_ showed an inactivation rate similar (*p* > 0.05) to the evolved strains, unlike Se_*∆flhA*_ (SeCarB and SeCarC) and Se_*wbaV*_, which showed an even higher tolerance to carvacrol than the evolved strains.

### Cross-tolerance to heat

**Fig. 4:**
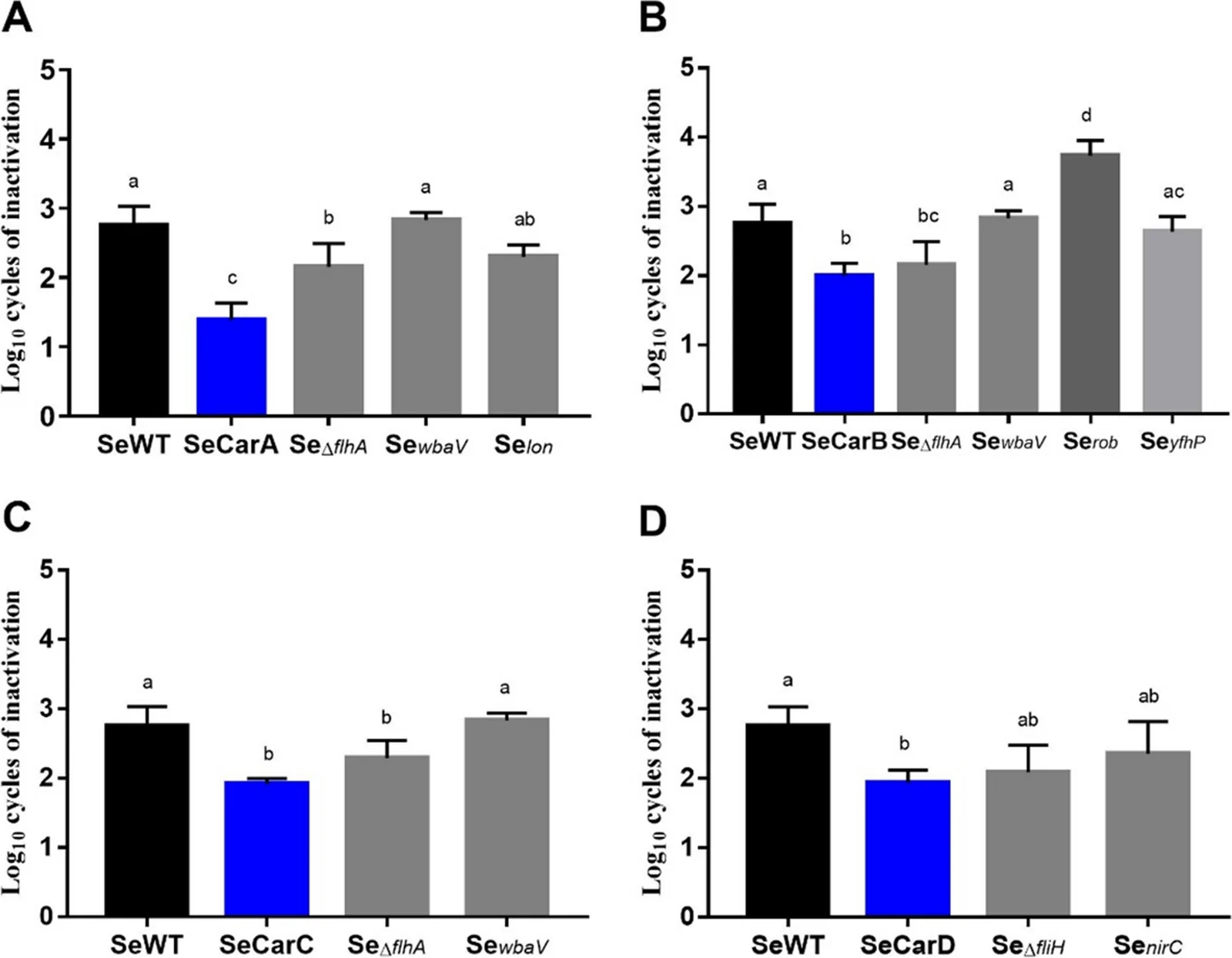
Log_10_ cycles of inactivation of *Salmonella enterica* Typhimurium LT2 (■; SeWT), the evolved strains (  ): SeCarA (A), SeCar (B), SeCarC (C), SeCarD (D). and the strains constructed with isolated genes (  ) after 25 min of heat treatment at 54 °C in McIlvaine buffer pH 7.0. Data are means ± standard deviations (error bars) obtained from at least three independent experiments. Different superscript letters represent statistically significant differences (*p* ≤ 0.05)

To evaluate the role of mutations in cross-tolerance to food processing methods, we characterized survival of the evolved and of the constructed strains to heat treatments. As can be seen in Fig. 4, after 25 min at 54 °C at pH 7.0, survival of the four evolved strains was approximately one log_10_ cycle higher than SeWT (*p* ≤ 0.05), thereby underscoring the cross-tolerance of evolved strains to heat.

We studied this increase in tolerance throughout all the constructed strains, and we observed that the majority of the single mutations (Se_*lon*_, Se_*nirC*_, Se_*yfhP*_, Se_*wbaV*_, and Se_*∆fliH*_) were not responsible for that increase, as they did not show significant differences compared with SeWT (*p* > 0.05) in terms of increased tolerance to heat. Moreover, the mutation in Se_*rob*_ even caused *Salmonella* to become more susceptible to heat than SeWT, since Se_*rob*_ was inactivated for one log_10_ cycle more than wild-type strain. However, the genetic variation found in *flhA* did seem to play a highly relevant role in the increased tolerance of the evolved strains to heat. Nevertheless, the level of tolerance displayed by SeCarA could not have been imposed on it by itself, but only by the simultaneous occurrence of all three mutations. Similarly, the simultaneous occurrence of mutations in *fliH* and *nirC* would be required in order to explain the higher tolerance of SeCarD as compared to SeWT.

### Cross-resistance to antibiotics

We likewise assessed the occurrence of cross-resistance to other antimicrobials, such as antibiotics for clinical use, in SeWT and its evolved strains. Table 5 displays the MIC to several antibiotics against them: ampicillin, cephalexin, ciprofloxacin, chloramphenicol, kanamycin sulfate, nalidixic acid sodium, rifampicin, tetracycline, and trimethoprim.

**Table 5:** Minimum inhibitory concentration (MIC; µL/L) of antibiotics: ampicillin (Amp), cephalexin (Lex), ciprofloxacin (Cip), chloramphenicol (Chl), kanamycin sulfate (Kan), nalidixic acid sodium (Nal), rifampicin (Rif), tetracycline (Tcy), and trimethoprim (Tmp) against *Salmonella* Typhimurium LT2 (SeWT) and the selected evolved strains (SeCarA, SeCarB, SeCarC, and SeCarD)

Strains	Antibiotics (µg/mL)								
	Amp	Cip	Chl	Kan	Lex	Nal	Rif	Tcy	Tmp
SeWT	1	0.016	4	4	8	8	16	4	1
SeCarA	2	0.016	8	4	8	8	16	4	1
SeCarB	2	0.032	16	2	8	16	32	8	2
SeCarC	1	0.016	8	4	4	8	16	4	1
SeCarD	1	0.016	4	4	8	8	16	4	1

As shown by the table, SeCarD strain did not show cross-resistance to any of the tested antibiotics in comparison to SeWT. Similar results were observed in SeCarC, which showed higher resistance to chloramphenicol, but lower resistance against cephalexin. In the case of SeCarA, a double increase in MIC was observed in both ampicillin and chloramphenicol; meanwhile, SeCarB showed cross-resistance to all the above-mentioned antibiotics, except for cephalexin and kanamycin. The influence of each of the identified mutations was verified and it was shown that the increased resistance against chloramphenicol in SeCarA and SeCarC was due to *wbaV*; *lon* caused the increased ampicillin resistance in SeCarA and *rob* was the responsible for all the increases observed in SeCarB.

## Discussion

In the past decade, the European Commission (Regulation (EC) No 1334/2008) and the US Food and Drug Administration (FDA 21CFR172.515 1977) have approved the use of several EOs and ICs in food products. These natural antimicrobials are also under study as potential alternatives to antibiotic treatments against multidrug-resistant bacteria. Initially, the development of microbial resistance against EOs was ruled out in view of their great complexity and compositional variety and, therefore, in view of the multitude of different antimicrobial action mechanisms that their ICs can exert on bacteria (Luz da Silva et al. 2012). However, ALEs showed that exposure to subinhibitory concentrations could lead to resistance and tolerance against complex EOs such as *Citrus sinensis or Thymbra capitata*, in *S. aureus* (Berdejo et al. 2020b) and *Listeria monocytogenes* (Berdejo et al. 2021b), respectively*.* Moreover, exposure to ICs such as carvacrol, citral, and limonene oxide can also favor the isolation of resistant variants against them, as occurs in *Escherichia coli* (Chueca et al. 2016) or *Staphylococcus aureus* (Berdejo et al. 2019).

Nevertheless, all those ALE studies evaluated one sole lineage; it is thus unknown whether the behavior and the genetic changes caused by the exposure of those microorganisms to EOs or ICs under selective pressure follow any sort of common or general pattern. We therefore decided to carry out an experimental design with four lineages of ALE against carvacrol; MIC determinations showed that those lineages had acquired varying degrees of resistance against it. Lineages A and B (SeCarA and SeCarB) showed an increase in MIC of carvacrol, as opposed to lineages C and D, which showed a degree of resistance comparable to SeWT. As the latter strains, SeCarC and SeCarD, had also been obtained after 10 steps under selective stress, we maintained them in the study in order to assess if any other kind of resistance might have occurred in them. To further characterize the evolved strains, growth kinetics in the presence and absence of carvacrol were determined. SeCarA and SeCarB showed an adaptation to the natural antimicrobial (shorter lag phase and higher growth rate) that did not come at a growth fitness cost in the absence of carvacrol (growth parameters were similar to SeWT). Similar results were described by Berdejo et al. (2020a), whose resistant variants of *S.* Typhimurium obtained in ALE with carvacrol demonstrated better fitness than the wild type under selective pressure. Although SeCarC and SeCarD showed similar MIC of carvacrol than SeWT, their lag phases (Table 2) in the presence of carvacrol were shorter than that of the SeWT. These results showed that these evolved strains were better adapted to carvacrol, although the adaptation mechanism did not modify their maximum growth rate in the presence of carvacrol (*µ*_*m*_). Regarding maximum absorbance (*A*), the differences observed among strains were not due to a different number of cells, as was verified by bacterial counts (CFU/mL), but to other unknown physiological characteristics such as shape, intracellular composition, etc.

Further characterizing the evolved strains’ tolerance to carvacrol, we found that lethal treatments showed a higher tolerance in all four evolved strains in comparison to SeWT. These results imply that the expected effectiveness of lethal treatments with carvacrol might not be sufficient to inactivate target pathogen bacteria when tolerant strains such as these emerge within the food chain, as was demonstrated by Berdejo et al. (2020a). Interestingly, increased tolerance to carvacrol was demonstrated even for SeCarC and SeCarD, which displayed the same MIC (resistance) as SeWT. These results are consistent with those published by Levin-Reisman et al. (2017) and by Sulaiman and Lam (2021), who suggested that tolerance to antimicrobials precedes and facilitates the evolution of resistance. Therefore, SeCarC and SeCarD might eventually become resistant if the ALE study was prolonged, since the evolution of tolerance can lead to the fixation of partial-resistance mutations that substantially elevate the probability of full resistance (Levin-Reisman et al. 2017). In addition, our results confirm that the emergence of resistant variants as a response to ALE with carvacrol did not follow a general pattern, at least as far as the observed strains are concerned, since each of the four lineages displayed a thoroughly individual behavior pattern in terms of resistance and tolerance.

In regard to genotypic characterization, WGS and Sanger sequencing revealed 10 mutations (Table 2), of which four were present in more than one strain, as was the case of *rrsH*, *sseG*, *wbaV*, and *flhA*, and six that were unique, that is, they were present in only one strain (*nirC*, *fliH*, *lon*, *rob*, upstream *yfhP*, and upstream *argR*).

Among all the genetic variations we had found, three were discarded from the study for the following reasons: (i) *rrsH* is the most widely used marker gene for profiling bacterial communities due to its hypervariability; it is thus probably unrelated to the increased resistance (Yang et al. 2016); (ii) *sseG*, whose SNV is a silent mutation; and (iii) the SNV found upstream *argR*, which was discarded because it was found in an intergenic area (STM4463–STM4464) and encoded a putative arginine repressor that has not been associated with resistance.

The seven remaining mutations found among the four lineages appear to be related to metabolic pathways and gene functions known from previous investigations, but those studies did not additionally fabricate single-mutation constructs in order to confirm the role of the affected genes in increasing bacterial resistance. We thus constructed the de novo strains featured in this study by substituting the WT alleles with the mutant ones in SeWT (Se_*lon*_, Se_*rob*_, Se_*nirC*_, Se_*wbaV*_, Se_*yfhP*_) and by deleting *flhA* and *fliH* (∆*flhA* and ∆*fliH*).

Phenotypic characterization of the constructed strains was carried out regarding the following.

### Resistance to carvacrol

Se_*lon*_ and Se_*rob*_ were the only constructed strains that showed an increased resistance against carvacrol in comparison to SeWT. The genetic modifications in *lon* and *rob* were responsible for the observed increased resistance against carvacrol, as Se_*lon*_ and Se_*rob*_ showed the same MIC as the evolved mutants SeCarA and SeCarB, respectively (Table 1). The *lon* gene encodes an ATP-dependent protease that plays the role of posttranslational quality control in bacterial cells, clearing misfolded proteins. Matange (2020) suggested that loss of that protease activity, depending on the concentration of the drug, can confer a selective advantage at low concentrations, but might be detrimental at high concentrations. Deletion of the *lon* gene leads to increased levels of MarA, which overactivates the efflux of AcrAB-TolC pump responsible for drug efflux (Frimodt-Møller and Løbner-Olesen 2019). This could be the mechanism that makes Se_*lon*_ more resistant to carvacrol, so that the SNV we observed would be causing a loss of protease function. In addition, *rob*, a transcriptional regulator, is another gene that is linked to MarA, as it activates its expression (Jain and Saini 2016). The observed increase in resistance and better fitness in the presence of carvacrol could be attributed to a rise in the activity of the drug efflux pump, which, caused by Rob and MarA activation, might remove carvacrol from cytoplasm (Duval and Lister 2013). It is noteworthy that Rob and MarA are structurally homologous with SoxS and belong to the AraC/XylS family, which has the ability to respond to multiple threats such as drugs and toxic compounds, acidic pH, host antimicrobial peptides, and oxidative stress (Duval and Lister 2013).

### Tolerance to carvacrol

All constructed strains (Se_*lon*_, Se_*rob*_, Se_*nirC*_, Se_*wbaV*_, Se_*yfhP*_, Se_*∆flhA*_, and Se_*∆fliH*_) may be independently responsible for increased tolerance against carvacrol: among them, Se_*wbaV*_ (SNV: T581C) shows the most significant effect. *wbaV* participates in the group of genes responsible for the biosynthesis of O antigen polysaccharides (LPS), which play an important role in bacterial virulence. Jaiswal et al. (2015) demonstrated that deletion of *wbaV* increased resistance against antimicrobial peptides, including polymyxin B, protamine, cecropin, and normal human serum. Similarly, Berdejo et al. (2021a) showed that a frameshift deletion in the same gene, which might lead to a non-functional protein, increased tolerance in an evolved *Salmonella* strain in the presence of *Thymbra capitata* EO, whose main IC is carvacrol. The mechanism behind that increased tolerance remains unclear, but the presence of LPS, the main component of the outer membrane, appears to be crucial for bacterial defense against carvacrol (Fig. 3). Our results suggest that if only the SNV in *wbaV* had occurred, the increased tolerance of the evolved strain against carvacrol could have been significantly higher (*p* < 0.05). However, this effect seems to be offset, except if the other mutations simultaneously occur. Our findings indicate that different mutations in various genes, each with distinct functions, lead to the same increased tolerance to carvacrol in various lineages.

### Heat (cross-)tolerance

The evolved strains (SeCarA, SeCarB, SeCarC, and SeCarD) showed cross-tolerance against heat and, as shown in Fig. 4, our results indicate that *flhA*, involved in flagellar biosynthesis, plays a fundamental role in increasing bacterial tolerance to heat. Interestingly, the function of *flhA* in protecting against other food preservation technologies remains unclear. Studies with *E. coli* also underscored the relationship between flagellar synthesis and tolerance to high hydrostatic pressure (Gayán et al. 2019); however, no study has previously linked the flagellum with heat tolerance. In contrast, certain authors have observed a negative correlation between flagellar expression and resistance to antibiotics and multidrug resistance regulators, the latter including MarA, SoxS, and Rob (Thota and Chubiz 2019). In this sense, Lyu et al. (2021) concluded that flagellar motility uses cellular energy stored in the form of proton motive force and makes cells less efficient in pumping out toxic molecules such as antibiotics or H_2_O_2_ (Karash et al. 2017). Our study has also identified a mutation in SeCarD affecting *fliH* related to the flagella. Berdejo et al. (2020a) previously reported a SNV in *fliG*, which encodes the FliG protein that is part of the complex located at the base of the basal body of the flagellum. Taken together, our findings indicate that bacterial motility is closely linked to bacterial resistance.

In addition, our study revealed that the same SNV in *rob* could affect bacterial defense in different ways, depending on the food preservation method applied. As shown in Table 1, *rob* is responsible for resistance to carvacrol, while Fig. 4 indicates that it makes bacteria more sensitive to heat. This finding highlights the complex and multifaceted nature of bacterial defense mechanisms and the importance of understanding the specific mechanisms of action of food preservation technologies.

### Antibiotic (cross-)resistance

Finally, mutations in *lon* and *rob* are responsible for cross-resistance to antibiotics. Specifically, the SNV in *lon* has been found to cause resistance to ampicillin and chloramphenicol. Lon-deficient *E. coli* cells can tolerate higher concentrations of several antibiotics, including tetracycline, kanamycin, and erythromycin, as compared to cells that produce Lon (Matange 2020). On the other hand, the SNV in *rob* has been found to confer resistance to most antibiotics, including ampicillin, ciprofloxacin, chloramphenicol, nalidixic acid, rifampicin, tetracycline, and trimethoprim, but not to kanamycin. Notably, this mutation makes SeCarB more sensitive than SeWT to kanamycin. Additionally, the SNV in *wbaV* was the responsible for doubling the MIC of chloramphenicol against the evolved strains SeCarA and SeCarC.

These findings illustrate that emerging mutants can acquire not only direct resistance to the antimicrobial agents used in evolution treatments but also cross-resistance to a wide range of antibiotics. This therefore highlights the importance of the genetic variations present in SeCarA, SeCarB, and SeCarC for the development and spread of AMR, which, in turn, could have significant implications for public health.

Although the SNVs identified in *nirC* and upstream *yfhP* do not appear to be directly responsible for increased resistance and tolerance to carvacrol, and neither for cross-resistance/tolerance to heat or antibiotics, they are still of interest. *nirC* encodes an integral membrane protein that transports nitrite and nitrate across the cytoplasmic membrane (Rycovska-Blume et al. 2015). Berdejo et al. (2020a) detected the same SNV in *nirC*, where its presence might play a role in the appearance of resistant variants in the course of carvacrol ALE. While the SNV located upstream of *yfhP* does not appear to affect resistance, its potential relevance to oxidative stress (Karash et al. 2017) and its possible relationship with bacterial response to EOs and ICs have both been noted (Chueca et al. 2018). In a genetic analysis of evolved *Escherichia coli*, mutations in the *acrR*, *marA*, and *soxR* genes were identified, and they were found to be associated with resistance and tolerance to ICs (Chueca et al. 2018). Moreover, in an evolved strain of *S.* Typhimurium, Berdejo et al. (2020a) identified a different SNV that affected the *soxR* gene, and this was confirmed to be the main cause of increased resistance and tolerance against EO, both in laboratory media and in food, as well as against antibiotics (Berdejo et al. 2021a).

In conclusion, both phenotypically and genotypically, there is no common pattern of mutagenesis under selective pressure of carvacrol, since different mutations in different genes occur in different lineages. We nevertheless observed a common trend in two different lineages (SeCarA and SeCarB) toward increasing their resistance to carvacrol. Furthermore, although the use of EOs and ICs in the agri-food industry has been proposed to prevent the spread of antimicrobial resistance, the observed bacterial cross-resistance/tolerance against heat treatments and antibiotics would question the efficacy of EOs and ICs in preventing the appearance and propagation of AMR. Further studies are nevertheless required to ascertain under what conditions cross-resistance against antibiotics can occur, and to further explore the potential impact and scope of resistant variants such as these in the agri-food industry and in the public health domain.

## Supplementary Information

Below is the link to the electronic supplementary material.

Supplementary file1 (PDF 309 KB)

## Data Availability

Our manuscript has associated data in a data repository, it has data included as electronic supplementary material, and data will be available on reasonable request.
