# Supplementary material for: Adaptive evolution of Salmonella Typhimurium LT2 exposed to carvacrol lacks a uniform pattern
Source: Appl Microbiol Biotechnol. 2024 Jan 4;108(1):38. doi: 10.1007/s00253-023-12840-6 (PMC10766787; doi:10.1007/s00253-023-12840-6)
Supplement: Supplementary file 1 — Supplementary file1 (PDF 309 KB) [file 253_2023_12840_MOESM1_ESM.pdf]

Journal name: Applied Microbiology and Biotechnology

Manuscript title: Adaptive evolution of *Salmonella* Typhimurium LT2 exposed to carvacrol lacks a uniform pattern

Authors' names: Elisa Pagán, Natalia Merino, Daniel Berdejo, Raúl Campillo, Diego García-Gonzalo, Rafael Pagán*.

Affiliations:

Departamento de Producción Animal y Ciencia de los Alimentos, Facultad de Veterinaria, Instituto Agroalimentario de Aragón-IA2 (Universidad de Zaragoza-CITA), Zaragoza, Spain

***Corresponding author**: Dr. Rafael Pagán (pagan@unizar.es; +34 976762675)

Table S1. Primers used for PCR amplification and Sanger sequencing to verify the mutations of strains evolved of *Salmonella* Thyphimurium LT2. F: Forward; R: Reverse.

Gene	F primer (5' → 3')	R primer (5' → 3')
flhA	GGCAGGAACCCCATATCCTG	GCCGGTCTGATTGGTGAAGA
fliH	TGTGGACGATCGCAGTATCC	CATCAGCCGTGAGGCGATAA
lon	AGTACCTTGATTCGCCAGCC	TGGCCCAACGAGGTTTTACC
nirC	CAGCCAACAAAGAGGCAGTG	ATAAACGCCAGCAGACACCA
wbaV	GCCATAACATGCCATAGCCA	GCAGTGATGATGCTCTTGCG
rob	ATAAAATCCTGCACGCCGGT	AAGTGGCACCTGCAGAGAAT
sseG	TGTTAACGCGCCTGAGGAAT	ACGTTGTTCTGGCGTTACCT
argR	GCCGCCGTATGGATAAGGAT	GTCGTAACCGCCTACCAGTC
yfhP	TTCATCCTTCAGACGACCGC	GTCAATATCGCACGGAACG
rrsH	GCAACGCGAAGAACCTTACC	TTTGATTCGTTTCCGGGCG

Table S2. Primers used to construct the new strains. F: Forward; R: Reverse.

Gene	Sense	Primer sequence
lon	**F**	CGAAGGCTACATCAAGCTGAACAAAAAAATTCCGCCGGAAGTGCTGACGTTCCTAATTTTTGTTGACACTCTATC
	**R**	CACGGCTTTGAACCGCGAGATACTCAAGGATGCGGTCTTTCACGCGCTCCATCAAAGGGAAAACTGTCCATATGC
yfhP	**F**	AAGTAACCGCGTCAATATCGCACGGAACGCTAAATACCTGACTAATTTACTCCTAATTTTTGTTGACACTCTATC
	**R**	GCAGGGACAAGTCTGACATTCCCGAGTAATTTGGTCAACTATTTACTTGAATCAAAGGGAAAACTGTCCATATGC
wbaV	**F**	ATATTTATGTGACAGAAAAGAGACCGGGTGTGATTTAGTTGAGATTAGAATCCTAATTTTTGTTGACACTCTATC
	**R**	CGTTTTAAAGATATATTTTTACTGTAAAAATCATTAGCTAATTTTAAATAATCAAAGGGAAAACTGTCCATATGC
nirC	**F**	TAAGTGTGCGGCTAACGCTGCGCGCATCGCACGCCTGTCGGCGAATAATCTCCTAATTTTTGTTGACACTCTATC
	**R**	TTGCCAGACAAACCAGCCAGTTACACAGCGCGCCTTTGAAGAACAGTACCATCAAAGGGAAAACTGTCCATATGC
rob	**F**	GTTTATCGAAATCAGCAGTGGCGCTGCGTTTGACCGCGCGTCCAATTCTGTCCTAATTTTTGTTGACACTCTATC
	**R**	CGGGATGCGCCGACTGAACATAGCCATCGGCCTGCTCTTGCGGCAGTGCGATCAAAGGGAAAACTGTCCATATGC
fliH	**F**	TCTGGCGGAAACCGGCGAAATGGTGATTGGCAGCGGCGAGGATACCTATGATTCCGGGGATCCGTCGACC
	**R**	CGAGATCGTCGGGGGTCCAGACTTGCCACGGCAATTCATTAGACATAGGTTGTAGGCTGGAGCTGCTTCG
flhA	**F**	CCTTCCGGTGCCAGAAGCGCTGGATTTTATGAACGAGAAGAATACTGATGATTCCGGGGATCCGTCGACC
	**R**	CAGGGGAAAGAGCAACAACGCCAGCCATTTACGCATTATTTTCCTCCAATTGTAGGCTGGAGCTGCTTCG

Table S3. *A* (maximum OD595). *µm* (maximum specific growth rate; h-1) and *λ* (lag time; h) values and error standard of the modified Gompertz model obtained from at least 3 independently growth curves of *Salmonella* Typhimurium LT2 wild type (SeWT) (A) and evolved strains: SeCarA (B). SeCarB (C). SeCarC (D) at different concentrations of carvacrol. The goodness of the fit is shown by R^2 and adjusted R^2 values and the root mean square error (RMSE).

A) SeWT

Carvacrol (µL/L)	Values			Standard error			Godness of fit		
	A	*µmax*	*λ*	*A*	*µmax*	*λ*	R^2	*Adj* R^2	*RMSE*
0	0.718	0.159	3.185	0.020	0.035	0.509	0.938	0.932	0.069
	0.639	0.174	2.987	0.017	0.036	0.414	0.946	0.940	0.056
	0.645	0.211	3.238	0.019	0.057	0.452	0.918	0.910	0.069
	0.751	0.138	2.741	0.021	0.025	0.526	0.936	0.930	0.070
100	0.573	0.154	5.718	0.008	0.017	0.227	0.986	0.985	0.028
	0.525	0.166	5.339	0.015	0.021	0.211	0.989	0.987	0.024
	0.573	0.131	5.279	0.005	0.008	0.148	0.995	0.995	0.017
	0.587	0.158	5.836	0.006	0.013	0.161	0.993	0.993	0.020

B) SeCarA

Carvacrol (µL/L)	Values			Standard error			Godness of fit		
	A	*µmax*	*λ*	*A*	*µmax*	*λ*	R^2	*Adj* R^2	*RMSE*
0	0.724	0.329	3.521	0.012	0.060	0.216	0.972	0.969	0.045
	0.782	0.179	2.938	0.020	0.037	0.473	0.942	0.936	0.071
	0.821	0.207	2.917	0.019	0.039	0.403	0.946	0.941	0.069
	0.829	0.137	2.410	0.023	0.023	0.537	0.938	0.932	0.074
100	0.569	0.390	4.559	0.005	0.052	0.096	0.991	0.990	0.020
	0.639	0.162	3.692	0.014	0.027	0.359	0.963	0.959	0.048
	0.503	0.347	4.853	0.005	0.037	0.086	0.993	0.992	0.018

C) SeCarB

Carvacrol (µL/L)	Values			Standard error			Godness of fit		
	A	*µmax*	*λ*	*A*	*µmax*	*λ*	R^2	*Adj* R^2	*RMSE*
0	0.834	0.170	2.314	0.026	0.040	0.597	0.911	0.902	0.090
	0.760	0.199	2.630	0.023	0.047	0.491	0.924	0.915	0.078
	0.821	0.161	2.021	0.024	0.033	0.567	0.909	0.901	0.085
	0.624	0.296	3.052	0.009	0.031	0.121	0.995	0.994	0.019
100	0.616	0.293	3.738	0.013	0.070	0.276	0.951	0.946	0.051
	0.623	0.361	3.473	0.015	0.118	0.292	0.934	0.927	0.058
	0.643	0.414	3.567	0.008	0.076	0.143	0.978	0.975	0.034

D) SeCarC

Carvacrol (µL/L)	Values			Standard error			Godness of fit		
	A	*µmax*	*λ*	*A*	*µmax*	*λ*	R^2	*Adj* R^2	*RMSE*
	0.841	0.201	2.817	0.015	0.027	0.309	0.970	0.967	0.052
0	0.809	0.214	3.134	0.017	0.039	0.365	0.959	0.955	0.061
	0.811	0.172	2.887	0.015	0.022	0.329	0.970	0.967	0.051
	0.612	0.232	3.861	0.014	0.052	0.324	0.956	0.951	0.051
100	0.635	0.166	3.373	0.018	0.040	0.493	0.931	0.924	0.065
	0.645	0.194	4.360	0.014	0.037	0.347	0.961	0.957	0.051

E) SeCarD

Carvacrol (µL/L)	Values			Standard error			Godness of fit		
	A	*µmax*	*λ*	*A*	*µmax*	*λ*	R^2	*Adj* R^2	*RMSE*
	0.791	0.370	3.210	0.022	0.132	0.419	0.902	0.893	0.089
0	0.814	0.306	3.350	0.021	0.080	0.377	0.939	0.933	0.077
	0.830	0.155	2.317	0.027	0.031	0.567	0.925	0.917	0.083
	0.647	0.275	4.300	0.009	0.041	0.190	0.985	0.984	0.032
100	0.642	0.209	3.959	0.010	0.031	0.246	0.977	0.974	0.038
	0.703	0.153	2.995	0.016	0.025	0.413	0.953	0.949	0.056
	0.704	0.177	3.314	0.015	0.031	0.373	0.957	0.952	0.055

Table S4. Genetic variations detected by whole genome sequencing (WGS) between SeWT and the reference genome of *Salmonella* Typhimurium LT2 (NCBI accession: NC_003197.2). Single nucleotide variation (SNV). insertion (Ins) and deletion (Del).

Genome position	Genes	Locus tag	Mutation*	Change	Information
**290.718**	*rrsH*	STM0249	SNV: C1529A	No coding	RNA 16S ribosomal
**364.623**	*crl*	STM0319	Del: -T 104	Frame shift	Sigma factor-binding protein
**416.555**	*prpR*	STM0367	SNV: C1159T	Leu387Phe	Operon regulator
**453.939**	*brnQ*	STM0399	SNV: C681T	Silent mutation (Tyr227)	Branched-chain amino acid transport system carrier protein
**509.118**	*cypD*	STM0452	SNV: T450A	Asp150Glu	Peptidylprolyl isomerase
**608.859**	*fimH*	STM0547	SNV: G182C	Gly61Ala	Adhesin
**1.205.933**	Intergenic *wraB - ycdF*	STM1119 STM1120	SNV: G →A	No coding	-
**1.778.104**	*ycjF*	STM1684	SNV: T821C	Leu274Pro	UPF0283 membrane protein
**1.841.398**	-	STM1747	SNV: G98A	Arg33Gln	Hypothetical protein
**1.849.642**	*hnr*	STM1753	SNV: T305G	Val102Gly	Regulator of RpoS
**3.469.143**	*dacB*	STM3300	SNV: C483T	Silent mutation (Ser161)	Transpeptidase
**3.673.628**	*malQ*	STM3513	SNV: T287G	Leu96Arg	4-Alpha-glucanotransferase
**3.675.952**	*malP*	STM3514	Del: -GCCGCCTG 358	Frame shift	Alpha-1.4 phosphorylase
**3.819.815**	-	STM3633	SNV: T562C	Silent mutation (Leu188)	LacI family transcriptional regulator
**4.122.937**	*gppA*	STM3913	SNV: G385T	Gly129Cys	Pyrophosphatase
**4.122.950**	*gppA* *rhlB*	STM3913 STM3914	Del: -1.179 pb	Knock-out (*gppA*. *rhlB*)	Pyrophosphatase ATP-dependent RNA helicase RhlB
**4.291.432**	*yiiQ*	STM4082	SNV: G323A	Stop-gain	Hypothetical protein
**4.294.693**	*glpK*	STM4086	SNV: G1171A	Asp391Asn	Glycerol kinase
**4.697.694**	*treB*	STM4454	Ins: + A 543	Frame shift	Pseudogene (trehalose metabolism)

*Position respect to the start of the coding region.
